# Extent of Traumatic Spinal Cord Injury Is Lesion Level Dependent and Predictive of Recovery: A Multicenter Neuroimaging Study

**DOI:** 10.1089/neu.2023.0555

**Published:** 2024-09-17

**Authors:** Simon Schading-Sassenhausen, Dario Pfyffer, Lynn Farner, Andreas Grillhösl, Orpheus Mach, Doris Maier, Lukas Grassner, Iris Leister, Armin Curt, Patrick Freund

**Affiliations:** ^1^ Spinal Cord Injury Center, Balgrist University Hospital, University of Zurich, Zurich, Switzerland.; ^2^ Department of Anesthesiology, Perioperative and Pain Medicine, Stanford University School of Medicine, Palo Alto, California, USA.; ^3^ Spinal Cord Injury Center, BG Trauma Center Murnau, Murnau, Germany.; ^4^ Department of Neurosurgery, Christian Doppler Clinic, Paracelsus Medical University, Salzburg, Austria.; ^5^ Wellcome Trust Centre for Neuroimaging, UCL Queen Square Institute of Neurology, University College London, London, United Kingdom.; ^6^ Department of Neurophysics, Max Planck Institute for Human Cognitive and Brain Sciences, Leipzig, Germany.

**Keywords:** biomarker, MRI, recovery prediction, spinal cord injury

## Abstract

Assessing the extent of the intramedullary lesion after spinal cord injury (SCI) might help to improve prognostication. However, because the neurological level of injury impacts the recovery potential of SCI patients, the question arises whether lesion size parameters and predictive models based on those parameters are affected as well. In this retrospective observational study, the extent of the intramedullary lesion between individuals who sustained cervical and thoracolumbar SCI was compared, and its relation to clinical recovery was assessed. In total, 154 patients with subacute SCI (89 individuals with cervical lesions and 65 individuals with thoracolumbar lesions) underwent conventional clinical magnetic resonance imaging 1 month after injury and clinical examination at 1 and 12 months. The morphology of the focal lesion within the spinal cord was manually assessed on the midsagittal slice of T_2_-weighted magnetic resonance images and compared between cervical and thoracolumbar SCI patients, as well as between patients who improved at least one American Spinal Injury Association Impairment Scale (AIS) grade (converters) and patients without AIS grade improvement (nonconverters). The predictive value of lesion parameters including lesion length, lesion width, and preserved tissue bridges for predicting AIS grade conversion was assessed using regression models (conditional inference tree analysis). Lesion length was two times longer in thoracolumbar compared with cervical SCI patients (F = 39.48, *p* < 0.0001), whereas lesion width and tissue bridges width did not differ. When comparing AIS grade converters and nonconverters, converters showed a smaller lesion length (F = 5.46, *p* = 0.021), a smaller lesion width (F = 13.75, *p* = 0.0003), and greater tissue bridges (F = 12.87, *p* = 0.0005). Using regression models, tissue bridges allowed more refined subgrouping of patients in AIS groups B, C, and D according to individual recovery profiles between 1 month and 12 months after SCI, whereas lesion length added no additional information for further subgrouping. This study characterizes differences in the anteroposterior and craniocaudal lesion extents after SCI. The two times greater lesion length in thoracolumbar compared with cervical SCI might be related to differences in the anatomy, biomechanics, and perfusion between the cervical and thoracic spines. Preserved tissue bridges were less influenced by the lesion level while closely related to the clinical impairment. These results highlight the robustness and utility of tissue bridges as a neuroimaging biomarker for predicting the clinical outcome after SCI in heterogeneous patient populations and for patient stratification in clinical trials.

## Introduction

Spinal cord injury (SCI) is a severe condition, which permanently impacts patients’ daily life. Patients usually suffer from sensorimotor impairments, autonomous dysfunction, and persistent pain in many cases. The extent of recovery is uncertain and underlies significant variability. Clinical examination after SCI follows a standardized protocol according to the International Standards for Neurological Classification of Spinal Cord Injury (ISNCSCI).^1,2^ Subsequently, patients are classified into five severity grades on the American Spinal Injury Association Impairment Scale (AIS), which represents the current gold standard assessment for the severity of SCI in clinical routine.^2^ However, despite its wide use and clinical utility, the AIS classification is insensitive to account for different trajectories of recovery that may occur within the same AIS grade.^3–6^ Implementing conventional magnetic resonance imaging (MRI) readouts into clinical practice to complement clinical measures might help to improve prognostication and was suggested to improve clinical decision-making.^7,8^

The intramedullary lesion site can be easily assessed on conventional MR images. Especially, the craniocaudal and anteroposterior extent of the lesion core—including injury-spared tissue bridges—can be reliably quantified at the subacute stage and, on its own, evaluate the injury severity and predict recovery following SCI.^9–14^ However, one important aspect to consider in these predictive models is the neurological level of injury (NLI). Thoracic SCI patients show the highest proportion of neurologically complete injuries and the worst recovery.^15^ On the other hand, patients with cervical injuries demonstrate higher conversion rates for AIS grades.^15,16^ A key question here remains whether the morphology and extent of the focal lesion appear differently at various spinal levels and how this relates to changes in AIS grades, as the NLI might considerably influence predictive models. Therefore, we investigated whether differences between cervical and thoracolumbar injuries are reflected in the morphology of the lesion and whether, despite these potential differences, conventional neuroimaging readouts of the lesion can add valuable information for outcome prediction.

Based on previous findings,^9,12,15^ we hypothesized that (i) the extent of the focal lesion is more severe in thoracolumbar compared with cervical SCI patients, (ii) AIS grade converters show less pronounced lesion parameters than nonconverters, independent of the NLI, and (iii) anteroposterior lesion parameters, in particular tissue bridges, help to improve predictions of AIS grade change from the subacute to the chronic phase, despite potential differences in the severity of the lesion among different NLI.

## Materials and Methods

### Participants and study design

This retrospective observational study comprises 154 subacute SCI patients (89 cervical and 65 thoracolumbar SCI patients) who were admitted consecutively to the rehabilitation program at the Balgrist University Hospital, Zurich, Switzerland (43 cervical and 30 thoracolumbar SCI patients) and the BG Trauma Center, Murnau, Germany (46 cervical and 35 thoracolumbar SCI patients) between July 2002 and February 2021. All participants underwent standard clinical MRI scanning including T_1_-weighted (T_1_-w) and T_2_-weighted (T_2_-w) scans of the spinal cord, approximately 1 month after the injury. Participants with concomitant brain injuries or intraaxial lesions were excluded. Further exclusion criteria were preexisting neurological or mental disorders, medical disorders leading to functional impairments, and contraindications to MRI. Moreover, patients were excluded if simultaneous cervical and thoracic lesions and/or spinal damage were observed. For 36 patients, the conversion status could not be determined because of missing follow-up clinical examinations 12 months after injury. Therefore, the final study cohort for conversion and prediction analyses consisted of 118 SCI patients in total, of which 69 were cervical and 49 were thoracolumbar SCI patients.

### Standard protocol approvals, registrations, and patient consents

All participants were included in the European Multicenter Study about Spinal Cord Injury and provided written informed consent before participation in the study, which was approved by the local Ethics Committee (EK-03/2004) and conducted in accordance with the Declaration of Helsinki.

### Clinical assessments

Participants underwent a comprehensive clinical examination protocol according to the ISNCSCI, which was conducted in the subacute phase at 1 month and in the chronic phase at 12 months after injury.^17^ This examination protocol included the assessment of lower extremity and upper extremity motor scores as well as the whole-body light touch score and pin prick score. Based on these assessments, patients were classified into categories A–E on the AIS, with grade A indicating a sensorimotor complete lesion and grade E no functional impairments.

### Image acquisition

MRI scanning was performed on clinical 1.5T and 3T scanners. The 1.5T MRI scanners included Siemens Avanto (or Avanto^fit^), Siemens Espree, Siemens Symphony, GE Signa HDxt, and Philips Achieva. The 3T scanners included Siemens Skyra (or Skyra^fit^) and Siemens Verio. The scanning protocol consisted of conventional clinical sagittal T_1_-w scans, sagittal T_2_-w scans, and transversal T_2_-w scans, which were obtained at the lesion site.

### Image analysis

**FIG. 1.**
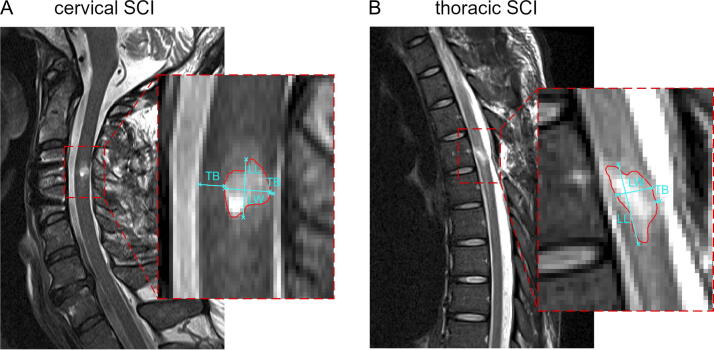
Quantitative assessment of lesion parameters on representative T_2_-weighted midsagittal images in a cervical SCI patient **(A)** and a thoracic SCI patient **(B)**. The outline of the intramedullary cystic cavity is indicated in red and the respective lesion parameters in light blue. SCI, spinal cord injury; LL, lesion length; LW, lesion width; TB, preserved tissue bridges width.

Focal tissue damage at the site of injury includes edema, inflammation, hemorrhage, and, finally, the formation of a cystic cavity, which manifests as a hyperintense signal on T_2_-w scans at 3–4 weeks post-SCI.^18,19^ The focal tissue damage was quantified on sagittal T_2_-w scans by segmenting the hyperintense area. Using the Jim software (V.9.0., Xinapse Systems), the lesion was identified on the midsagittal slice and manually delineated by three raters (S.S.-S., D.P., and L.F.) who were blinded to patient identity. This manual segmentation includes the craniocaudal lesion parameter lesion length, the anteroposterior parameters lesion width, and the total width of preserved tissue bridges as the sum of ventral and dorsal tissue bridges (Fig. 1). Tissue bridges represent injury-spared tissue with a low signal intensity between the hyperintense focal lesion and the surrounding spinal canal.^9^

### Statistical analysis

Statistical analyses were conducted using RStudio (Version 4.0.5). Differences between cervical and thoracolumbar SCI were tested using unpaired two-tailed *t*-tests for age at injury and the time interval between injury and scanning time point, as well as clinical examination time points, chi-squared test for sex and AIS grade at baseline, and Cochran–Mantel–Haenszel test for AIS grade conversion. A significance threshold of *p* = 0.05 was used.

Agreement between lesion segmentation raters was assessed based on the Zurich data set by calculating the two-way random effects intraclass correlation coefficients (ICCs) for all lesion parameters (i.e., lesion length, lesion width, and tissue bridges).

To determine differences between cervical and thoracolumbar SCI patients as well as AIS subgroups, an analysis of variance (ANOVA) was created for all three lesion parameters with AIS grade (four levels: A–D) and NLI (two levels: cervical and thoracolumbar SCI) as independent variables. Similarly, ANOVA was used to investigate differences between cervical and thoracolumbar SCI patients that improve in their AIS grade (converter) and those that do not improve (nonconverter). The lesion parameters were used as dependent variables, whereas conversion status (two levels: converter and nonconverter), NLI (two levels: cervical and thoracolumbar SCI), and AIS grade (four levels: A–D) were included as independent variables. A post-hoc pairwise comparison with Tukey’s correction (*p* < 0.05) was used to identify significant group differences.

Finally, unbiased recursive partitioning conditional inference tree (URP-CTREE) analysis was conducted for all three lesion parameters for prospective stratification of SCI patients into separate subgroups of recovery. This algorithm uses a set of predictors to split a relatively heterogenous patient population into more homogenous subgroups according to a predefined end-point. The splitting decisions try to maximize the discrepancy between the final subgroups. Here, the AIS grade at baseline (four levels: A–D), the NLI (two levels: cervical and thoracolumbar SCI), and the MRI lesion parameters (lesion length, lesion width, and tissue bridges) were used as predictor variables and the AIS grade at the 12-month follow-up as the end-point. Thus, this URP-CTREE analysis aims at finding homogenous subgroups of the 12-month AIS grade based on clinical and imaging parameters at 1 month after SCI.

### Data availability

The authors certify that they have documented all data, methods, and materials used to conduct the research presented. Anonymized data pertaining to the research presented will be available on request from investigators.

## Results

### Patients’ characteristics and clinical outcomes at baseline

**FIG. 2.**
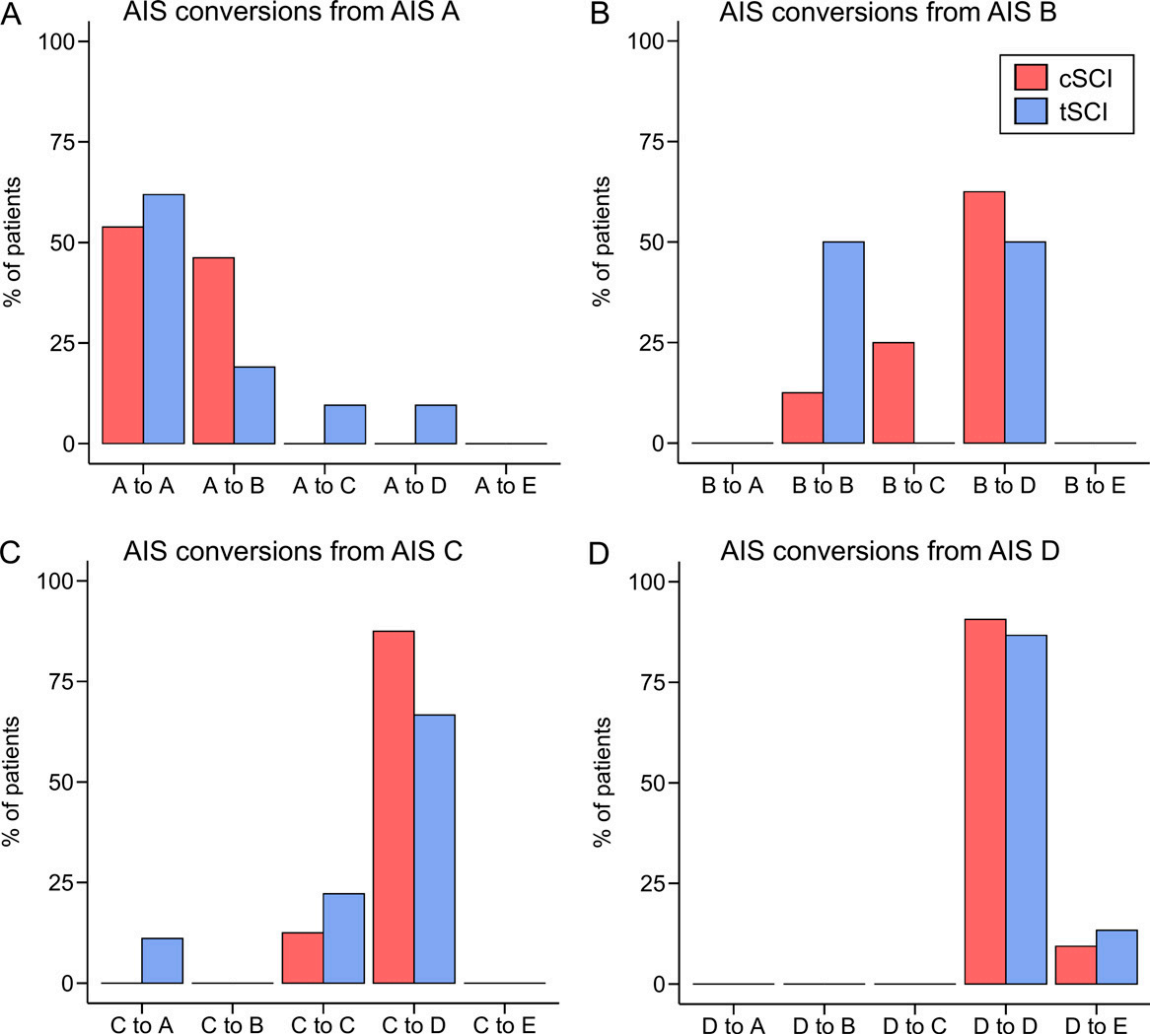
Comparison of American Spinal Injury Association Impairment Scale (AIS) grade conversion between cervical and thoracolumbar SCI patients for **(A)** AIS A, **(B)** AIS B, **(C)** AIS C, and **(D)** AIS D patients separately. cSCI, cervical spinal cord injury; tSCI, thoracolumbar spinal cord injury.

Clinical and demographic information of all SCI patients and the comparison between cervical and thoracolumbar SCI patients are listed in Table 1. The time between injury and both clinical and MRI examinations was not significantly different between cervical and thoracolumbar SCI patients. However, the groups differed in classification according to the AIS grade (*p* = 0.004). Overall, the conversion rates, accounting for different AIS grades, were higher in cervical than in thoracolumbar SCI patients, but this difference was not statistically significant (odds ratio for AIS conversion in cervical vs. thoracolumbar SCI: 1.73, *p* = 0.34) (Fig. 2).

**Table 1.** Clinical and Demographic Information of Cervical and Thoracolumbar Spinal Cord Injury (SCI) Patients

	Cervical SCI	Thoracolumbar SCI	*p* Value
Age ± SD [years]	50.9 ± 17.7	44.9 ± 17.7	0.043
Sex [male/female]	76/13	48/17	0.11
1-month clinical examination ± SD [days]	18.8 ± 10.7	18.6 ± 11.5	0.92
12-months clinical examination ± SD [days]	336.1 ± 88.2	330.5 ± 99.8	0.76
1-month MRI examination ± SD [days]	29.5 ± 19.3	28.6 ± 18.0	0.77
AIS score at 1 month [no.]			0.004
A	16	29	
B	10	6	
C	21	12	
D	42	18	

AIS, American Spinal Injury Association Impairment Scale.

### Agreement between lesion segmentation raters

The ICC of the inter-rater agreement between all three raters was moderate for lesion length (0.73), lesion width (0.72), and tissue bridges width (0.56).^20^ When comparing only the segmentations of the experienced raters (S.S.-S. and D.P.), the respective ICCs were as follows: lesion length (0.69), lesion width (0.83), and tissue bridges width (0.72), indicating that segmentation of anteroposterior parameters can be considerably improved by training. For further analysis, the segmentation results of rater S.S.-S. were considered.

### Difference in lesion parameters between cervical and thoracolumbar SCI patients

**FIG. 3.**
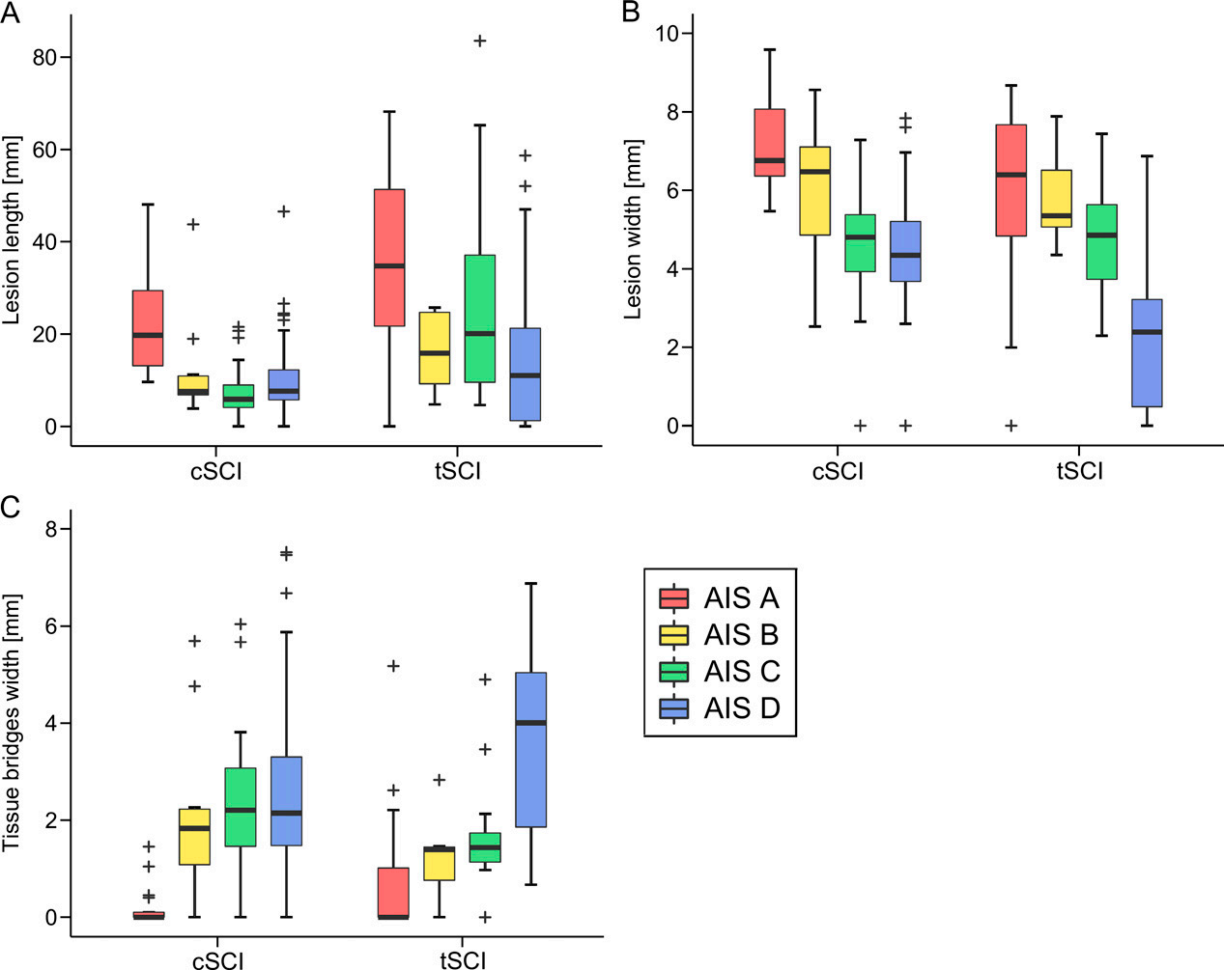
Comparison of lesion parameters at 1 month after injury between cervical and thoracolumbar SCI patients according to American Spinal Injury Association Impairment Scale (AIS) grade. **(A)** Lesion length, **(B)** lesion width, and **(C)** preserved tissue bridges width. Cervical and thoracolumbar SCI patients are subdivided according to the AIS grade at 1 month after injury. cSCI, cervical spinal cord injury; tSCI, thoracolumbar spinal cord injury.

By comparing lesion parameters at 1 month, we found a significant difference in lesion length between cervical and thoracolumbar SCI patients (F = 39.48, *p* < 0.0001), independent of AIS grade (Fig. 3A). A post-hoc test revealed that the lesion length was more than twice as long in thoracolumbar as in cervical SCI patients (cervical SCI: 12.20 mm; thoracolumbar SCI: 27.13 mm; difference: −14.94 mm, 95% confidence interval [CI]: −19.64 to −10.24 mm, *p* < 0.0001). In contrast, lesion width (F = 0.36, *p* = 0.55) and tissue bridges width (F = 1.25, *p* = 0.27) were not significantly different between both groups (Fig. 3B, C).

For all three lesion parameters, we found a significant effect of AIS grade on the respective lesion parameter (lesion length: F = 8.74, *p* < 0.0001; lesion width: F = 23.45, *p* < 0.0001; and tissue bridges width: F = 19.76, *p* < 0.0001). Post-hoc tests revealed that this effect was mainly driven by differences between SCI patients classified as AIS A and AIS D (Table 2), with AIS A showing the most severe lesion parameters (i.e., highest lesion length and lesion width, and smallest tissue bridges), whereas AIS D patients showed the least severe lesion measures (i.e., smallest lesion length and lesion width, and greatest tissue bridges).

**Table 2.** Comparison of Lesion Parameters Lesion Length, Lesion Width, and Tissue Bridges Width between Different AIS Categories

Parameter	Comparison	Difference	95% CI	*p* Value
Lesion length	A–B	13.04	2.02 to 24.06	0.013
	A–C	11.46	2.78 to 20.14	0.004
	A–D	13.06	5.59 to 20.52	<0.0001
	B–C	−1.58	−13.11 to 9.95	0.98
	B–D	0.02	−10.63 to 10.67	1.00
	C–D	1.60	−6.61 to 9.80	0.96
Lesion width	A–B	0.57	−0.82 to 1.96	0.71
	A–C	1.88	0.78 to 2.97	<0.0001
	A–D	2.79	1.85 to 3.74	<0.0001
	B–C	1.30	−0.15 to 2.76	0.096
	B–D	2.22	0.88 to 3.57	0.0002
	C–D	0.92	−0.12 to 1.95	0.10
Tissue bridges width	A–B	−1.20	−2.37 to −0.03	0.043
	A–C	−1.47	−2.40 to −0.55	0.0003
	A–D	−2.25	−3.04 to −1.45	<0.0001
	B–C	−0.27	−1.50 to 0.96	0.94
	B–D	−1.05	−2.18 to 0.09	0.082
	C–D	−0.77	−1.65 to 0.10	0.10

AIS, American Spinal Injury Association Impairment Scale.

### Difference in lesion parameters between converters and nonconverters

**FIG. 4.**
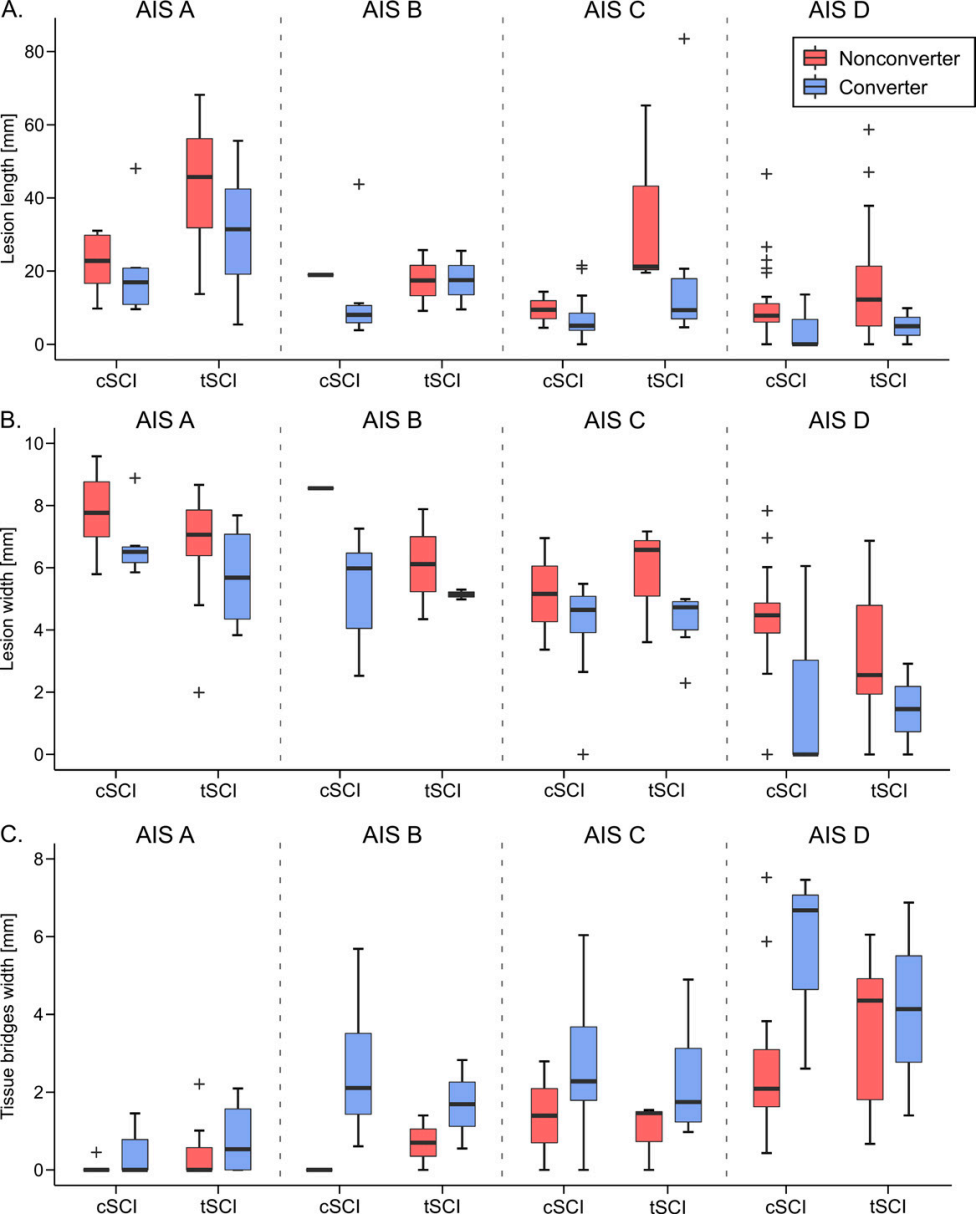
Comparison of lesion parameters at 1 month after injury between cervical and thoracolumbar SCI patients according to American Spinal Injury Association Impairment Scale (AIS) grade conversion between 1 and 12 months after injury. **(A)** Lesion length, **(B)** lesion width, and **(C)** preserved tissue bridges width. Cervical and thoracolumbar SCI patients are subdivided according to AIS grade at baseline. Conversion is defined as improvement of at least 1 AIS grade. cSCI, cervical spinal cord injury; tSCI, thoracolumbar spinal cord injury.

All three lesion parameters showed a significant main effect (i.e., independent of NLI and AIS grade at baseline) for conversion between 1-month AIS grade and 12-month AIS grade (lesion length: F = 5.46, *p* = 0.021; lesion width: F = 13.75, *p* = 0.0003; and tissue bridges width: F = 12.87, *p* = 0.0005) (Fig. 4). Post-hoc tests comparing the mean lesion parameters between converters and nonconverters averaged across all AIS grades at baseline showed that tissue bridges width was almost 1.5 times larger in converters than in nonconverters (converters: 2.25 mm; nonconverters: 1.87 mm; difference: 0.82 mm, 95% CI: 0.26 to 1.38 mm, *p* = 0.004). Similarly, lesion length (converters: 15.60 mm; nonconverters: 20.18; difference: −5.08 mm, 95% CI: −10.39 to 0.23, *p* = 0.061) and lesion width (converters: 4.65 mm; nonconverters: 5.03 mm; difference: −0.99 mm, 95% CI: −1.64 to −0.34 mm, *p* = 0.003) were lower in converters, however, with smaller relative differences between both groups.

### Stratification of SCI patients into recovery profiles using lesion parameters

**FIG. 5.**
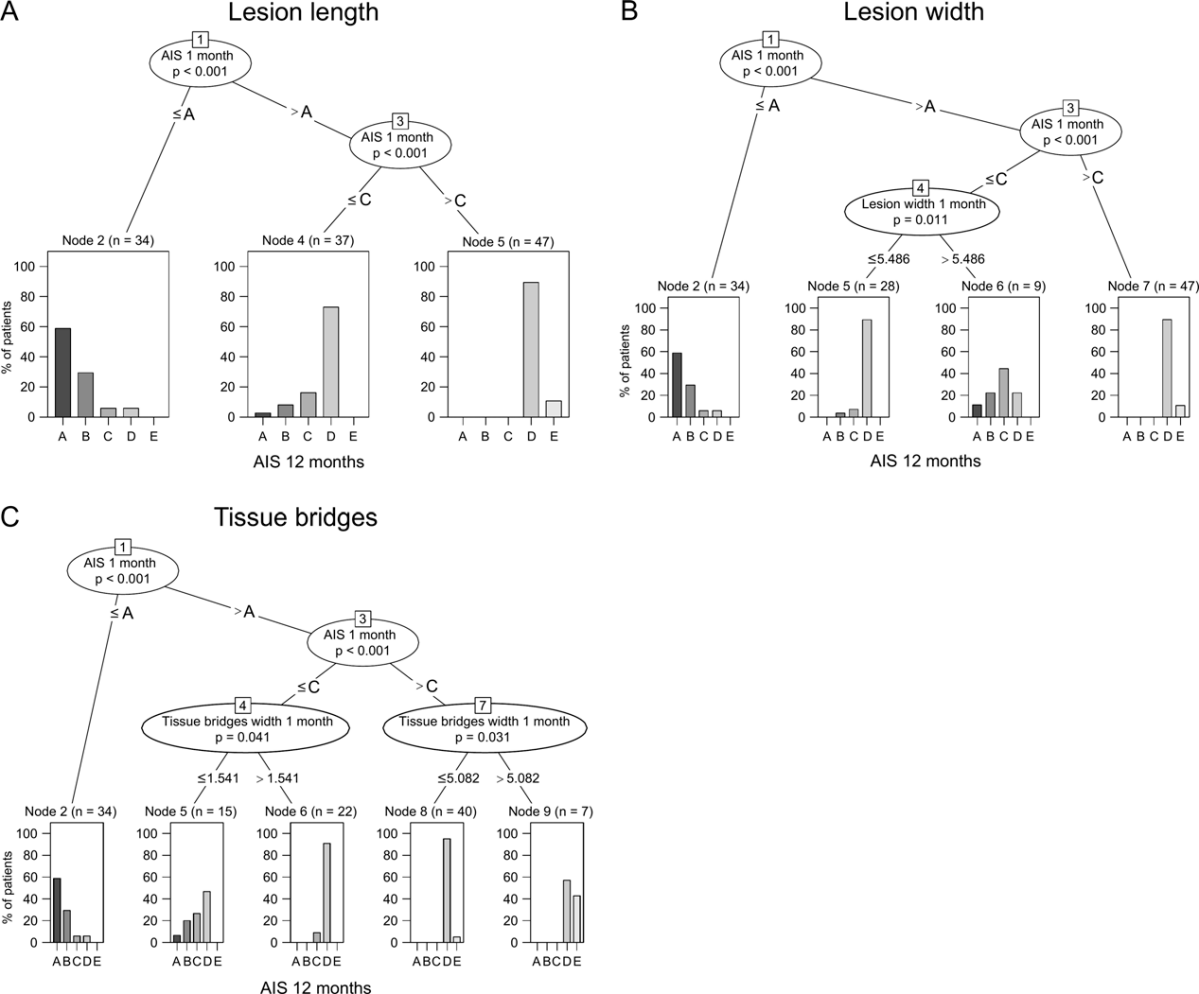
Unbiased recursive partitioning conditional inference tree analysis for the three lesion parameters lesion length **(A)**, lesion width **(B)**, and tissue bridges width **(C)**. The models included American Spinal Injury Association Impairment Scale (AIS) grade at 1 month after injury, the level of injury (cervical or thoracolumbar spinal cord injury), and the respective lesion parameters as predictors and the AIS grade at 12 months after injury as the clinical end-point variable. Every node represents a significant splitting decision for maximizing the discrepancy of the final subgroups. The distribution of AIS grades in the final subgroups is shown as bar graphs at the bottom. The reported *p*-values are corrected for multiple testing.

URP-CTREE analysis was used for subgrouping SCI patients according to their recovery profiles based on clinical assessments and imaging parameters at 1 month after injury. We found evidence that anteroposterior lesion parameters, in particular tissue bridges width, add relevant information for predicting the clinical outcome at 12 months after SCI (Fig. 5). In all three URP-CTREE models, the AIS grade at baseline was identified as a crucial parameter for splitting the patient population into more homogenous subgroups. In detail, the first splitting node separated patients with AIS A from the remaining AIS grades (node 1, AIS A vs. AIS B–D, *p* < 0.001) with AIS A patients having mostly AIS A and AIS B at the 12-month follow-up. The next node split the remaining AIS B–D patients according to their AIS score at baseline into the groups AIS B–C and AIS D (node 3, AIS B–C vs. AIS D, *p* < 0.001), where the subgroup AIS D showed mostly AIS grades D and even AIS grade E at the 12-month follow-up. Although these three subgroups could not be further subdivided by including the lesion parameter lesion length, both anteroposterior parameters lesion width and tissue bridges width added valuable information to these models for further subgrouping of SCI patients. A lesion width with a cutoff of 5.49 mm (node 4, *p* = 0.011) and tissue bridges width with a cutoff of 1.54 mm (node 4, *p* = 0.041) both identified a subgroup of AIS B–C patients with a higher percentage of conversions to AIS D. The classification accuracy for this subgroup was considerably higher for tissue bridges (node 6, error: 9.1%, Fig. 5C) and lesion width (node 5, error: 10.7%, Fig. 5B) compared with the final node in the model with lesion length (node 4, error: 27.0%, Fig. 5A). In addition, tissue bridges width with a cutoff of 5.08 mm (node 7, *p* = 0.031) could further subdivide AIS D patients into those that would more likely convert to AIS E and those that would rather stay at AIS D (node 8, error: 5.0%, Fig. 5C), thereby improving the classification accuracy of the terminal node in the lesion length model (node 5, error: 10.6%, Fig. 5A) and lesion width model (node 7, error: 10.6% Fig. 5B).

## Discussion

This study investigated whether conventional neuroimaging-derived lesion parameters (i.e., morphology of the lesion) in terms of lesion length, lesion width, and tissue bridges width differ between cervical and thoracolumbar SCI patients. It further assessed the association between these lesion parameters and neurological recovery in terms of AIS grade conversion and patient stratification into subgroups of recovery-specific profiles. We found that although the craniocaudal parameter “lesion length” was two times greater in thoracolumbar than in cervical SCI patients, the anteroposterior parameters “lesion width” and “tissue bridges width” were more related to neurological recovery. Spared tissue bridges, in particular, improved patient stratification and prediction of neurological recovery, independent of the NLI. These findings demonstrate the predictive potential of neuroimaging biomarkers based on conventional MRI, despite potential differences between cervical and thoracolumbar SCI patients, and highlight their potential value for patient stratification following SCI.

The first important observation in this study is that only specific aspects of the lesion differed between cervical and thoracolumbar SCI patients. Although the intramedullary lesion length was more than two times greater in thoracolumbar than in cervical SCI patients, there was no difference in the anteroposterior lesion parameters lesion width and tissue bridges width. It was previously shown that more severe injuries, both in animal SCI models and in comparison between AIS groups in human patients, cause a greater lesion extent in the craniocaudal direction than less severe injuries.^13,21,22^ In this study, more thoracolumbar SCI patients presented with AIS category A than cervical patients, whereas more cervical SCI patients were assessed as AIS D than thoracolumbar patients. Considering the anatomy and the biomechanics of the vertebral column, the thoracic segment is relatively stiff and stable because of support by the rib cage and the nearly vertically oriented articulating processes, whereas the cervical region is very mobile with a large range of motion.^23,24^ Therefore, one could argue that greater mechanical forces and an impact with higher energy are needed to produce injuries at the thoracic spine, which could explain the greater lesion length measured in thoracolumbar compared with cervical SCI patients.^15,25^ However, all models were corrected for AIS grade, suggesting that even within AIS categories, thoracolumbar patients showed a significantly greater lesion length. Further explanations for this difference between cervical and thoracolumbar SCI patients might be the anatomy of the vertebral canal and the perfusion of the spinal cord. First, the thoracic vertebral canal is relatively narrow, which might make it more susceptible to compressive forces.^15^ Second, the thoracic spinal cord is located in a watershed region of spinal perfusion, which predisposes this region for reduced blood supply during injuries.^26^ These factors could contribute to secondary injury processes and the expansion of the focal lesion along the cord.

However, in contrast to lesion length, lesion width and tissue bridges seem to be less influenced by the NLI and potential differences in trauma mechanism, anatomy of the vertebral canal, and perfusion of the spinal cord. Here, the question remains: which properties of the spinal cord and pathophysiological mechanisms cause this discrepancy between anteroposterior and craniocaudal lesion parameters? Previously, it was shown that secondary enlargement of the lesion, with its magnitude being related to the mechanical stress on the spine, occurs predominantly in the craniocaudal direction along the mechanically weakest parts of the spinal cord.^13,27^ This supports our findings that the difference in trauma mechanisms between cervical and thoracolumbar SCI mainly affects the lesion in the craniocaudal extent but not the anteroposterior extent. Being less sensitive to the influence of different trauma mechanisms, anteroposterior lesion parameters are more likely to be reliable prognostic markers for a heterogenous patient population.

Next, we assessed the prognostic potential of these lesion parameters by first comparing patients that converted in the AIS grade to a higher category and those patients not showing improvements in the AIS grade. For all three lesion parameters, converters showed significantly less severe outcomes than nonconverters. The most striking difference could be observed for tissue bridges width, which was almost 1.5 times larger in converters than in nonconverters. This suggests that tissue bridges might be more reliable biomarkers for identifying patients who will improve their functional outcome. This hypothesis is supported by various studies, which identified that the predictive potential of neuroimaging markers based on the lesion extent in the transversal plane, such as the BASIC score^28^ or tissue bridges width,^12^ might exceed neuroimaging markers measuring the craniocaudal extent of the lesion.^29–31^ From a pathophysiological point of view, anteroposterior lesion parameters represent surrogate markers for the amount of spared afferent and efferent fiber tracts within the spinal cord, which were shown to be crucial factors for recovery after SCI.^32^ Consequently, the superiority of these anteroposterior parameters, in particular tissue bridges width, highlights once more the importance of these preserved fibers and that the exact anatomical location and focal extent of the lesion are crucial to assess the amount and type of spared fiber tracts.^33–35^ It suggests that radiological assessments of the severity of a spinal trauma should focus on specific neural components that are relevant for recovery (i.e., spared fiber tracts).^32^

To assess and compare the predictive value of lesion parameters, URP-CTREE models were used for stratification of a relatively heterogenous patient cohort into more homogenous subgroups according to the clinical outcome at 12 months after injury. Early stratification of SCI patients is crucial for individualized therapies and the efficient planning of prospective clinical studies.^36^ These models suggest the superiority of anteroposterior lesion parameters for outcome prediction. Both, lesion width and tissue bridges width, split SCI patients into more homogenous subgroups, whereas lesion length did not add valuable information to the baseline clinical score for further subgrouping of SCI patients. Crucially, tissue bridges allowed the highest degree of stratification, which speaks to their potential as powerful variables for prediction-based stratification.

Moreover, these URP-CTREE models demonstrated once again the importance of clinical examination for prognostication. The AIS grade was identified as an important splitting decision variable in all three models (i.e., including either lesion length, lesion width, or tissue bridges width), which could afterwards be further subdivided using radiological parameters. First, AIS A patients were separated from the remaining AIS categories since these sensorimotor-complete SCI patients did not recover considerably and mostly stayed in AIS category A or showed minimal conversion. This splitting decision most likely reflects differences in the extent of spontaneous recovery between complete (i.e., AIS A) and incomplete SCI patients, where complete SCI patients show significantly lower rates of neurological recovery and AIS conversion.^16,37,38^ However, no lesion parameter could split the AIS A patient cohort into further subgroups to discriminate between patients that will stay in AIS A and those that will convert to different AIS categories, pointing out remaining heterogeneities that cannot be explained by the lesion morphology alone. The next decision rule in all three URP-CTREE models separated AIS D patients from AIS categories B–C. This AIS B–C patient cohort could be further subdivided using both anteroposterior lesion parameters (i.e., tissue bridges width and lesion width), where these parameters could identify one subgroup with a higher conversion to AIS D at 12 months after SCI. Compared with the model without additional splitting of the AIS B–C patient cohort, the classification accuracy considerably improved. Furthermore, using tissue bridges width as a predictor, in the remaining AIS D patient cohort, those patients with greater potential for converting to AIS E could be identified, reducing the classification error of the remaining AIS D cohort approximately by a factor of 2.

This analysis confirms the hypothesis and previous findings that radiological surrogate markers of preserved fiber tracts are more predictive of functional recovery and can improve patient stratification, given the importance of intact nerve fibers for recovery.^32^

The potential of measuring preserved tissue bridges on sagittal T_2_-w images for predicting neurological and functional recovery has already been demonstrated in several studies.^9,39,40^ Moreover, by separating ventral and dorsal tissue bridges, this measure allows to assess motor and sensory recovery separately.^12,35,41^ Here, we complement these findings and show that although thoracolumbar and cervical SCI patients might differ in the severity of the initial trauma, injury mechanism, and their potential for recovery, measuring spared fiber tracts using conventional MRI is sensitive for predicting the clinical outcome. Crucially, tissue bridges retained their prognostic value when correcting for the NLI and initial AIS grade, which points to their clinical utility for application in a heterogenous patient cohort. This becomes particularly important with the evidence that prognosis and recovery are influenced by the injury level, although the injuries might appear similarly.^15,42^

This study has some limitations. We only assessed lesion parameters based on the midsagittal slice, which limits the information about fiber tracts running more laterally and the ability to detect parasagittal lesion parts.^11^ Future studies should consider taking into account the 3-dimensional extent of the lesion and its exact location within the spinal cord for a better understanding of the affected fiber tracts. In addition, there was a significant difference in the distribution of AIS grades between cervical and thoracolumbar SCI patients, which might reflect differences in the biomechanics and injury mechanisms. However, to show that predictive models are robust despite this difference, the AIS grade was included as a covariate in all statistical models, which minimized potential confounding effects. Moreover, the focal lesion develops very dynamically over the first few weeks after injury, with the most drastic changes occurring during the first 48 h.^43^ This might influence the accuracy of prognostic models based on the assessment of the focal lesion and should be taken into account. However, in this study, we analyzed MRI scans during the subacute phase with an average time interval between injury and MRI scan of approximately 29 days, when the extent of the focal lesion has stabilized. Finally, the sample size for this study was relatively small, which could in particular affect subgroup analyses. However, since the data for this study were sampled from conventional clinical MRI scans of two separate SCI centers, the study population is representative of regular clinical practice and comparable to different observational data.^44–46^

## Conclusion

In conclusion, these findings demonstrate that specifically the length of the spinal cord lesion area is more extended in thoracolumbar than in cervical SCI patients in contrast to lesion width and extent of preserved tissue bridges. However, the latter measures were related to clinical recovery profiles. Therefore, conventional neuroimaging biomarkers of the lesion area and spared fiber tracts have the potential to improving prediction and patient stratification after injury and may be explored as surrogate markers of treatment interventions.

## Transparency, Rigor, and Reproducibility

This observational study was carried out retrospectively without preregistration using available imaging data that were acquired during clinical routine. Images of 208 patients were screened, of which 154 were included in the final analysis. The remaining patients were excluded because of missing clinical data at the specified time points, bad image quality caused by metal artifacts that made the assessment of the lesion impossible, or simultaneous cervical and thoracic lesions in the cord, which would confound the statistical analysis. Image analysis was performed by independent raters blinded to the clinical status of the patients. All equipment and software used to perform imaging and image processing are widely available from commercial sources. The key inclusion criteria and outcome evaluations are established standards. The authors agree to publish the article using the Mary Ann Liebert Inc. “Open Access” option under an appropriate license.
